# Characterization of the cecal microbiome composition of Wenchang chickens before and after fattening

**DOI:** 10.1371/journal.pone.0225692

**Published:** 2019-12-05

**Authors:** Zhen Tan, Lilong Luo, Xiaozhe Wang, Qiong Wen, Lu Zhou, Kebang Wu

**Affiliations:** Laboratory of Tropical Animal Breeding, Reproduction and Nutrition, College of Animal Science and Technology, Institute of Tropical Agriculture and Forestry, Hainan University, Haikou, P.R. China; University of Illinois, UNITED STATES

## Abstract

The cecum of poultry harbors a complex and dynamic microbial community which plays important roles in preventing pathogen colonization, detoxifying harmful substances, nutrient processing, and harvesting of the ingestion. Understanding and optimizing microbial communities could help improve agricultural productivity. In this study, we analyzed the composition and function of cecal microbiota of Wenchang chicken (a native breed of Bantam) before and after fattening, using high throughput sequencing technology. High-throughput sequencing of the 16S rRNA genes V3-V4 hypervariable regions was used to characterize and compare the cecal microbiota of Wenchang chicken before fattening (free-range in hill) and after fattening (cage raising). Sixteen phyla were shared by the 20 samples. Firmicutes and Bacteroidetes were the top two abundant phyla being 80% of the total microbiota. Samples of chickens prior to fattening were more dispersed than those after fattening. Twenty four microbes could be considered as biomarkers and 3 phyla revealed differences by variance analysis which could distinguish the two groups. Cecal microbiota in the before fattening group had higher abundance of functions involved in digestive system and biosynthesis of other secondary metabolites. The composition and function of cecal microbiota in Wenchang chicken before and after fattening under the two feeding modes, free range in hillside and cage raising, were found to be different. These results can be attributed to the differences in feeding modes and growth stages. In-depth study on the functions and interactions of intestinal microbiota can help us in developing strategies for raising Wenchang chickens and provide valuable information for the study of microbiota in the chicken gut.

## Introduction

The intestinal microflora plays physiological, nutritional, and immunological roles in maintaining the gut health of the host [1]. In consideration of food safety and public health [2, 3], intestinal microbiota of livestock and poultry has been extensively studied [4, 5]. Increased knowledge on the community structure and functional capacity of the gut microbiota can help discover the relationships between microbial functions and the host physiology and metabolism.

Cecal microbiota has the most complex microbial community in the chicken digestive tract which plays important roles in preventing pathogen colonization, detoxifying harmful substances, absorbing additional nutrients, nitrogen recycling from uric acid, producing essential amino acids, and digestion of non-starch polysaccharides (NSPs) [6]. Fermentation of NSPs leads to the production of short chain fatty acids (SCFAs) that are resorbed through the mucosa and catabolized by the host, contributing substantially to animal nutrition and inhibition of acid-sensitive pathogens [7]. From the day of hatching until 60 weeks of age, the cecal microbiota of egglaying hens could be defined as four different stages [8]. And only one day of exposure is enough for hens to transfer gut microbiota to the newly hatched chickens [9]. The dominating microbiota in cecum are the phyla Firmicutes, Bacteroidetes, Actinobacteria, and Proteobacteria [10]. The bacterial communities of the left and right cecum are similar [11]. Functional metagenomics analysis have revealed enrichment of sequences corresponding to carbohydrate metabolism [12].

Studies on the correlation between poultry cecum microflora and feed energy extraction efficiency have found that a significant number of different bacteria were found in birds with high and low apparent metabolizable energy extraction capabilities [13]. Whole genome sequencing of chickens combined with 16S rRNA gene sequencing of intestinal microbiota identified two cecal microbial taxa, *Methanobrevibacter* and *Mucispirillum schaedleri*, which were significantly correlated with fat deposition of chickens[14]. The abundance of *Methanobrevibacter* was positively correlated with the abdominal fat content of chickens and no affect the change of body weight. There was a negative correlation between *M*. *schaedleri* abundance and abdominal fat accumulation and body weight.

In the recent times, with the improvement of consumption consciousness, free range and organic chickens are favored more. The meat of outdoor chickens contain more protein than the indoor chickens [15]. By comparing the composition and function of cecum microbiota of Dagu chicken, found that the proportion of Bacteroides was lower in cage raising, and the ratio of Firmicutes/Bacteroidetes was higher. In free-ranging mode, higher abundance of cecum microbiota involved in amino acid synthesis and carbohydrate metabolism pathway than cage ranging [16].

In this study, we worked on Wenchang chicken, a local breed of Hainan, China, which is famous for its excellent meat quality. The breeding of Wenchang chickens is divided into three stages: raising chicks, raising chickens (free range), and fattening chickens (caged). The chickens (aged 42–120 days) are mainly raised on the traditional hillsides, and feed on natural grass, wild vegetables, insects and minerals, additional supplement the full-price commodity diet. At the age of 120 days, in order to further enhance the flavor, fat content, and quality of the meat, the chickens are fattened in cages. Under this feeding mode, the whole production process is divided into two management systems and the variation of intestinal microbiota also plays an important role in this process. Exploring the impact of intestinal microbiota under different growth and fattening stages of Wenchang chickens has important practical significance for enhancing the competitiveness of their products, and hence the economic benefits.

Studies on the microbial structure and function of the composition and function of cecal microbiota of Wenchang chickens have not been conducted. We characterized the composition and function of cecal microbiota of Wenchang chickens and explored the differences of cecal microbiota between the growth and fattening periods. Additionally, the breeding patterns of hillside free-range and intensive cage raising were also compared here which might provide insights for designing high efficiency feed formula and developing applicable probiotics to alter the intestinal microbial community to improve the growth of chickens and regulate chicken meat quality.

## Materials and methods

### Animals and sample collection

In the present study, Wenchang chickens of 120 days were collected from Longquan Wenchang Chicken Industrial co., Ltd. (Wenchang, Hainan, China). Birds were raised from 120 days of age to 180 days of age in cages (50 cm × 50 cm × 50 cm, 80 cm above ground). The house temperature was maintained at 23°C. The chickens were provided access to feed commodity diet and water ad libitum.

Ten chickens were randomly selected before cage raising (120 days old, group CC1) and at the end of fattening (180 days old, group CC2) period, respectively. Selected chickens were slaughtered by the way of bloodletting outside the neck and the digesta samples from the cecum were collected under aseptic conditions within 15 min. The samples were snap-freezed in liquid nitrogen. All the samples were collected in sterile tubes and stored in liquid nitrogen and then used for DNA extraction and PCR amplification. The difference in body weights between the two groups was significant (*P* >0.05) (S1 Table).

All the animal experiments were reviewed and approved by the Institutional Animal Care and Use Committee of Hainan University and were performed in accordance with the Guidelines for Experimental Animals of the Ministry of Science and Technology (Beijing, China). All the methods were in accordance with the guidelines approved by the Quality Supervision, Inspection, and Quarantine of the People’s Republic of China (GB/T 17236–2008). Client-owner consent was obtained to collect the samples from the Wenchang chickens.

### 16S rRNA gene sequencing

The microbial genomic DNA was extracted from the samples and purified using the QIAamp DNA stool mini kit (Qiagen Ltd., Germany) following the manufacturer’s instructions. Adequate quantity of high-quality genomic DNA was extracted, and the concentration of DNA was measured using a UV–Vis spectrophotometer (NanoDrop 2000c, USA). The V3-V4 region of the 16S rRNA gene was amplified by PCR with the universal bacterial 16S rRNA gene PCR amplicon primers (338F-806R, forward primer, 5'- ACTCCTACGGGAGGCAGCA-3'; reverse primer, 5'- GGACTACHVGGGTWTCTAAT-3') combined with adapter sequences and barcode sequences. PCR amplification was performed in a total volume of 50 μl, which contained 10 μl bμffer, 0.2 μl Q5 high-fidelity DNA polymerase, 10 μl high GC enhancer, 1 μl dNTP, 10 μM of each primer, and 60 ng genome DNA. Mixed PCR products were purified using the GeneJET Gel Extraction Kit (Thermo Scientific, USA) following the manufacturer’s instructions. High-throughput sequencing analysis of bacterial rRNA genes was performed on the purified, pooled sample using the Illumina Hiseq 2500 platform (2×250 paired ends) at Biomarker Technologies Corporation, Beijing, China. The data has been deposited in the National Center for Biotechnology Information’s Short Read Archive under accession no. SRP 230265.

### Sequence assembly and clustering

Paired-end reads from the original DNA fragments were merged using FLASH (version 1.2.7) [17]. Paired-end reads (tags) were assigned to each sample according to the unique barcodes and raw tags were quality controlled by Trimmomatic (version 0.33) [18]. High quality tag sequences were obtained by truncating the first low quality (Phred score < 20) base sites, filtering out the tags of continuous high-quality base length less than three quarters of the whole tags, and removing the chimeric sequences by UCHIME (version 4.2) [19]. Sequences with ≥ 97% similarity were assigned to the same operational taxonomic units (OTUs), picked by USEARCH (version 10.0) [20]. A threshold of 0.005% of all sequence numbers was used for filtering [21].

### Community annotation and taxonomy

Representative sequences of OTUs were annotated with taxonomic information from the reference database (Silva) [22] of microorganisms using Ribosomal Database Project (RDP) Classifier (version 2.2). The corresponding biological classification information of each OTU was obtained and then the composition of each sample community in each level (phylum, class, order, family, genus, species) was counted.

### Diversity analysis and functional prediction

Alpha diversity index was evaluated by Mothur (version 1.30) [23] and beta diversity analysis was performed by QIIME software (version 1.8.0) [24] which uses the four algorithms—binary jaccard, bray curtis, weighted unifrac, and unweighted unifrac to calculate the distance between samples [25]. Beta diversity analysis based on these four distance matrices mainly includes the following points [26]. Principal coordinate analysis (PCoA) was used to observe the difference of the sample population through the dimensionality reduction analysis of eigenvalues and eigenvectors. Non-MetricMulti-Dimensional Scaling (NMDS) analysis is based on monotone function data column replacement. Unweighted Pair-group Method with Arithmetic Mean (UPGMA) analysis was used to analyze the difference between samples based on the difference of evolutionary information between the different sample sequences. Line Discriminant Analysis (LDA) Effect Size (LEfSe) analysis was used for identifying biomarkers with statistical difference between the different groups [27]. Multivariate statistical test was performed using analysis of variance (ANOVA) [16].

The functions of all the OTUs were predicted by Kyoto Encyclopedia of Genes and Genomes (KEGG) and Clusters of Orthologous Groups of proteins (COG) databases, based on the structure of the gastrointestinal microbiota established using PICRUSt [28]. At the genus level, the species abundance of different groups were tested for the significant difference using paired t-test, and the *p* value threshold was 0.05 (<0.05 means significant).

## Results

### OTU clustering and taxonomic composition

**Fig 1:**
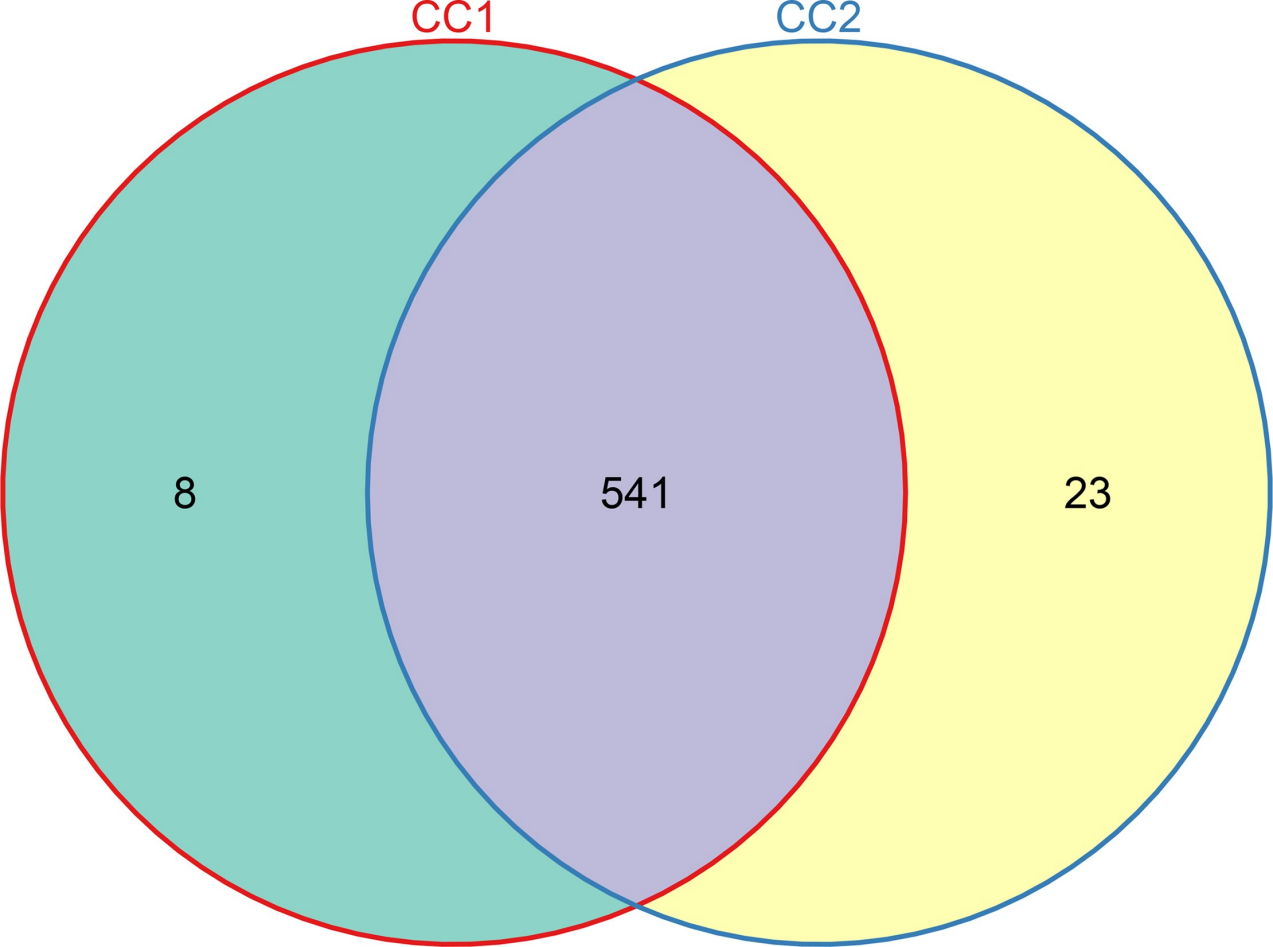
Shared OTU analysis of the different groups. CC1, cecal microbiota of the before fattening group; CC2, cecal microbiota of the after fattening group.

After quality control, demultiplexing, and assembly, from 55,277 to 66,164 valid tags were generated from each sample with a cut-off of 97% similarity by QIIME. Five hundred and forty nine OTUs were obtained in the cecal microbiota of the before fattening group (CC1) and 564 OTUs were observed in the after fattening group (CC2). A total of 541 OTUs were clustered in both sets of groups (Fig 1).

**Fig 2:**
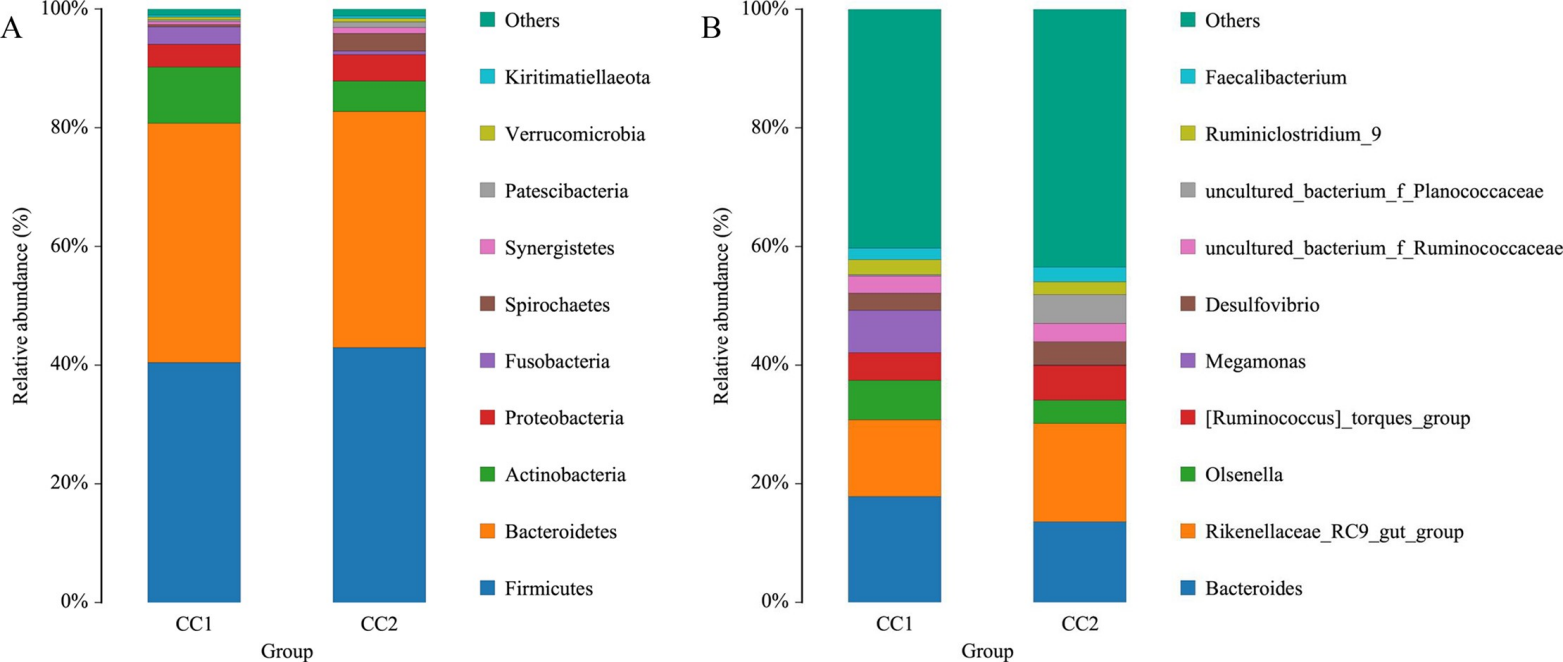
Histogram of the top 10 phyla (A) and genera (B) in each group.

Firmicutes, Bacteroidetes, Actinobacteria, and Proteobacteria were the top four phyla, regardless of the groups, and more than 90% of the sequences could be assigned to these 4 phyla (Fig 2A). Firmicutes and Bacteroidetes accounted for 40% of all the sequences in both the groups, respectively. In addition, the proportion of Actinobacteria was 9.46% in the CC1 group and 5.12% in the CC2 group. Proteobacteria accounted for 3.85% and 4.46% in CC1 group and CC2 group, respectively, Fusobacteria accounted for 2.94% and 0.59% in the two groups, and Spirochaetes were 0.38% and 2.98%, respectively.

At the genus level, top ten bacteria in both the groups accounted for 60% of the total reads (Fig 2B). The dominant *Bacteroides* and *Rikenellaceae_RC9_gut_group* together accounted for 30% of the total genera.

**Fig 3:**
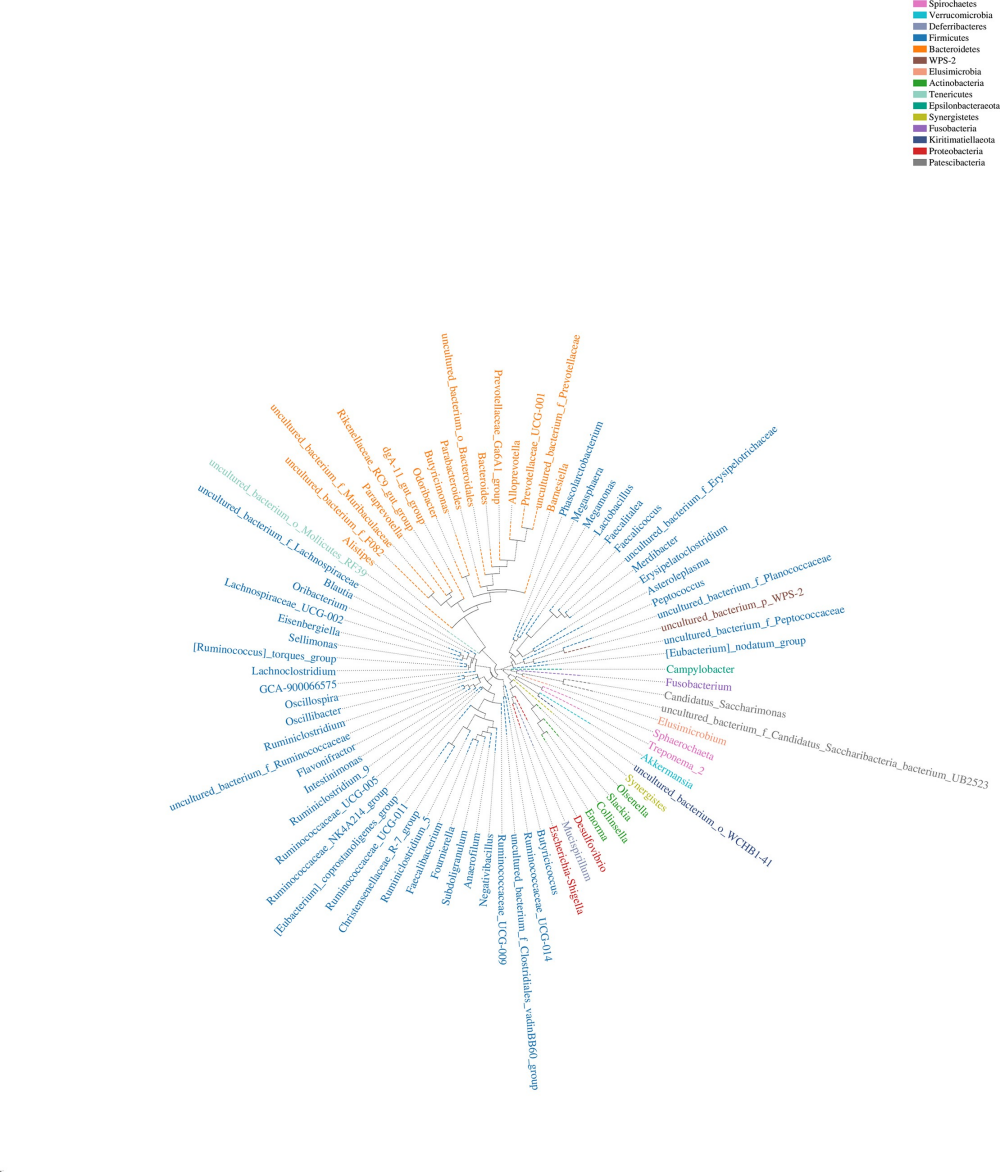
Phylogenetic tree of OTU at the genus level. The ring shows the phylogenetic tree, with the same color of the genus name representing the phyla.

Phylogenetic tree based on OTU sequence was constructed and a graph was drawn (Fig 3). In the evolutionary tree, each branch was represented as one species, and the length of the branch was the evolutionary distance between the two species which could be regarded as the degree of difference between the two species. Multiple genera in the evolutionary branches belonged to Firmicutes, and the genus *Akkermansia* in the Verrucomicrobia was also shown in the evolutionary branches.

### Variation in cecal microbiota of Wenchang chickens before and after fattening

**Fig 4:**
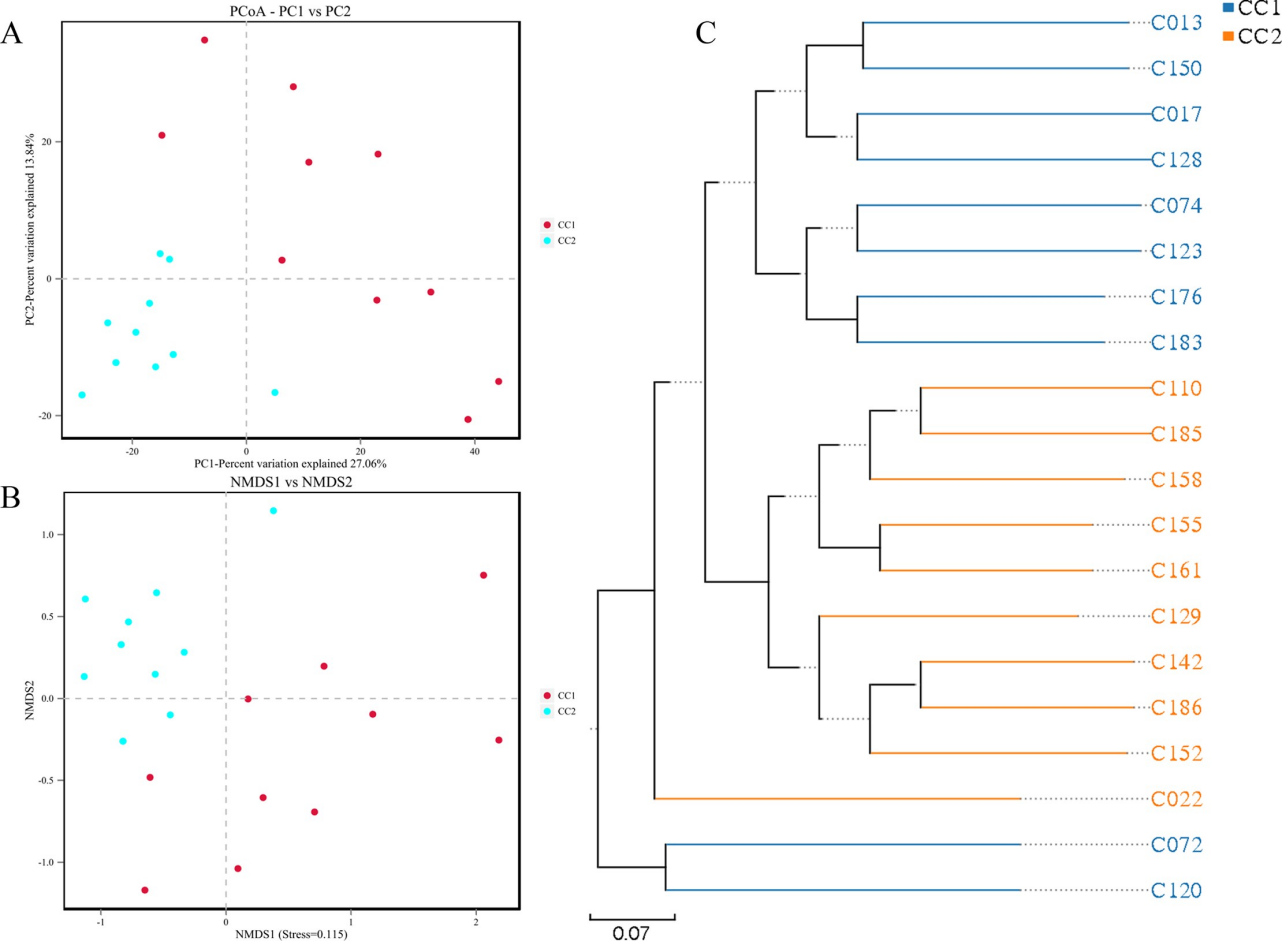
**PCoA analysis (A), NMDS ordination (B), and UPGMA cluster (C).** Principal coordinate analysis (PCoA), non metricmulti dimensional scaling (NMDS) plots, and unweighted pair-group method with arithmetic mean (UPGMA) phylogenetic tree demonstrate that cecum harbor different bacterial communities before and after the fattening stages.

PCoA revealed the variation between microbiome profiles based upon Bray-Curtis dissimilarity (Fig 4A). Coordinate 1 representing 27.1% of the variation, was associated with a different fattening period. Despite the lack of homogeneity in dispersion, the NMDS plot based on Bray-Curtis dissimilarity matrix still showed differences in microorganism distributions between the two groups (Fig 4B). The microorganisms in the CC1 group concentrated on one group whereas those in the CC2 groups concentrated on another.

To determine the degree of similarity among the samples, UPGMA was constructed based on Bray-Curtis distances (Fig 4C). Samples in the CC2 group were clustered into one major cluster, and except two samples, samples of the group CC1 were also together.

**Fig 5:**
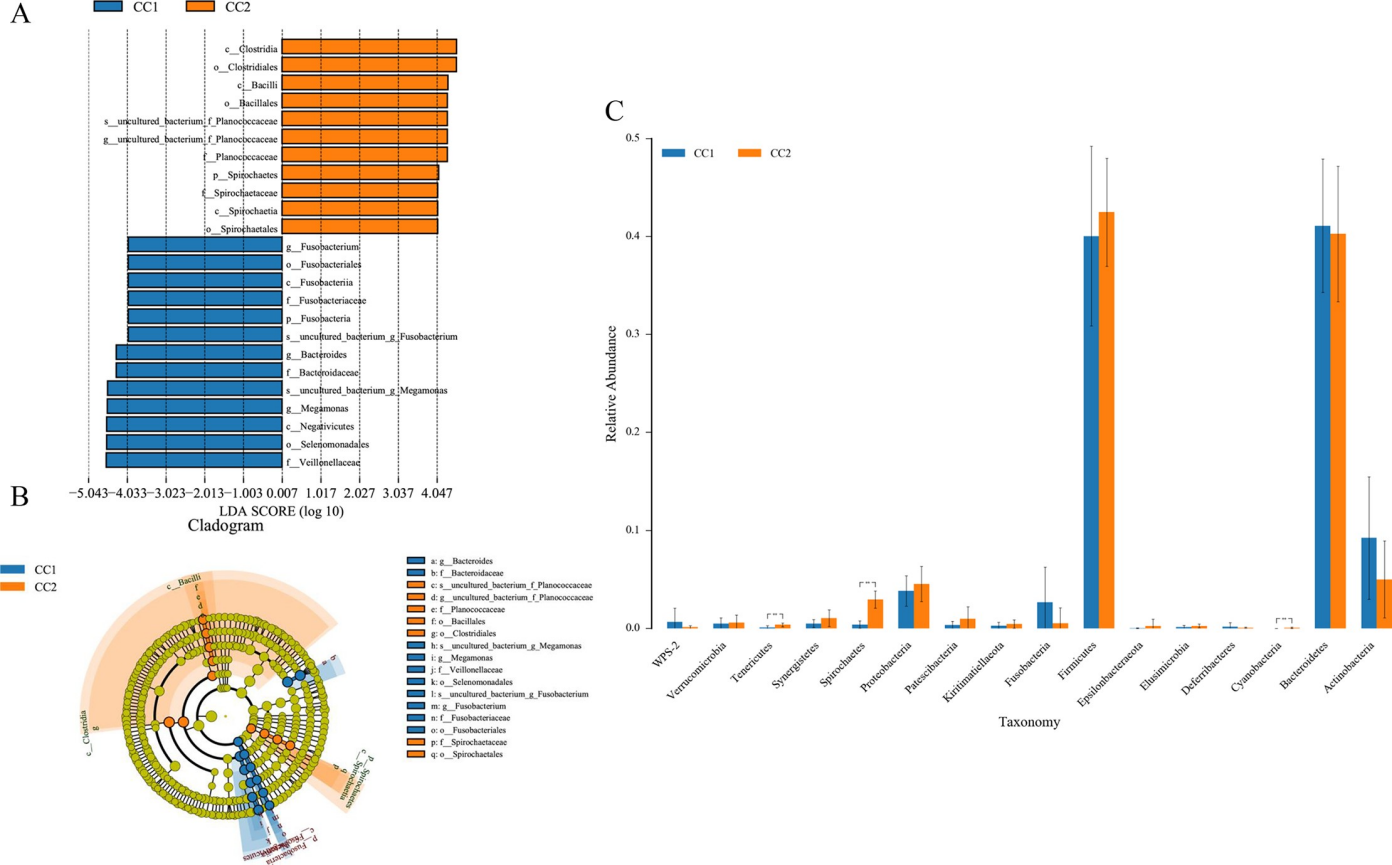
Significantly different microbes and the cladogram by LEfSe analysis (A and B), histogram of different phyla analyzed between groups by ANOVA (C).

The significant difference analysis was mainly used to find biomarkers with statistical differences among the different groups. In the cecum, 24 different taxonomic levels of microorganisms were found as potential biomarkers by LEfSe analysis for distinguishing between the before and after fattening groups (Fig 5A). In the microbial cladogram, the significantly different microorganisms were mainly classified as phylum Fusobacteria in the CC1 group and class Bacilli in the CC2 group, respectively (Fig 5B). A total of 16 phyla were shared between the groups and three significantly different (*P* < 0.05) phyla in the CC2 group were found to be Cyanobacteria, Spirochaetes, and Tenericutes by ANOVA (Fig 5C).

### Differences of microbial function between the before and after fattening groups

**Fig 6:**
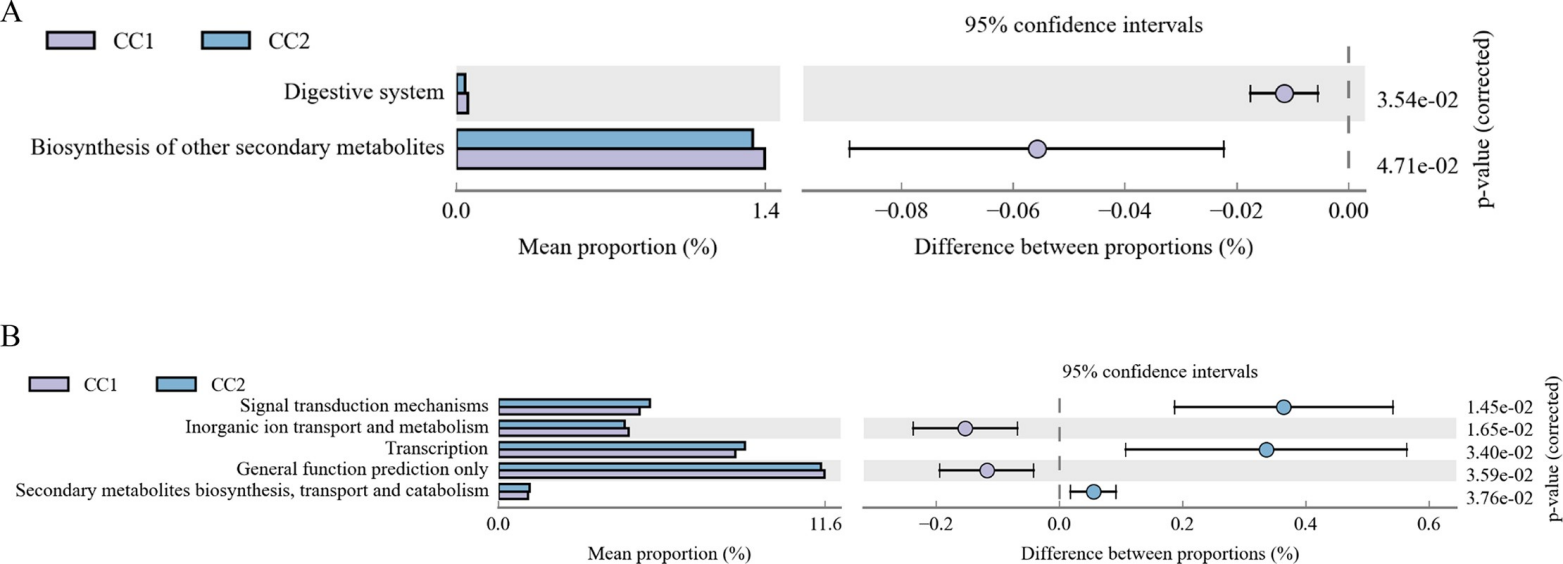
**Comparison of KEGG metabolic pathways between groups (A) and classification statistics of COG function (B).** The proportion of functional abundance differences within the 95% confidence interval. KEGG, Kyoto Encyclopedia of Genes and Genomes; COG, Clusters of Orthologous Groups of proteins.

A total of 43 second-level classification KEGG pathways were verified based on the structure of the cecal microbiota established using PICRUSt. “Carbohydrate metabolism”, “Global and overview maps”, and “Amino acid metabolism” were the top three functional annotations in both the groups, followed by “Energy metabolism” and “Metabolism of cofactors and vitamins”. The statistically significant second-level KEGG pathways of each comparison were identified by the paired t-test. “Biosynthesis of other secondary metabolites” and “Digestive system” significantly differed among the groups (Fig 6A).

Compared with COG, 25 second-level classifications were obtained in both the groups and the top annotations were “General function prediction only”, “Transcription”, “Carbohydrate transport and metabolism”, and “Amino acid transport and metabolism”. Five annotations which significantly differed between the groups were-“Transcription”, “Inorganic ion transport and metabolism”, “General function prediction only”, “Signal transduction mechanisms”, and “Secondary metabolites biosynthesis, transport and catabolism” (Fig 6B).

## Discussion

A diverse microbial community harbored in the intestinal tract closely interacts with the host. many factors influence the composition of gut microbiota, such as diet [29], host genetics [14, 30], medication [31], and rearing conditions [16]. Changes in feeding modes also greatly affect the poultry gut microbiota diversity, composition, and community structure. Significant differences in the cecum microbiota in Dagu chicken appeared under free range and cage raising feeding modes [16]. Different breeds of chickens affect the structure of intestinal microbiota and the flavor of chickens, which might be caused by the different diets and feeding modes [32].

Studies have found that Firmicutes and Bacteroidetes are the dominant bacteria in cecum, accounting for 80% of all the microbiota [10, 14, 16, 33, 34]. The phyla Firmicutes and Bacteroidetes dominate the intestines of mammals [35], which have been associated with SCFA metabolism. Firmicutes contribute to butyrate and propionate synthesis and Bacteroidetes primarily synthesize propionate, α-amylase, α-1,2-mannosidase, and endo-1,4-β-mannosidase [36] and are more likely to break down starch and other polymeric substances.

Increased fiber intake of outdoor free-range chickens directly affects the structure of gut microbiota, increases the abundance of Bacteroides and decreases the ratio of Firmicutes/Bacteroidetes [16]. In this study, the proportion of Firmicutes increased slightly after fattening, while the proportion of Bacteroidetes remained the same. The Wenchang chickens were kept in cages during the test, and the dietary fiber was not as abundant as in the free range mode. Meanwhile, as more energy absorbed, a high ratio of Firmicutes/Bacteroidetes also promoted obesity [7]. Numerous microorganisms in the large intestine, especially those in the cecum participate in microbial fermentation. The composition of these microorganisms was also closely related to fat deposits in the large intestine [37]. Caged fattening is usually included to make up for the hillside free range where there is weight and fat content reduction [38] and increases a certain amount of fat improving the quality of chicken.

Before and after fattening, the number of OTUs of cecal microbiota in the two groups were similar, and most of them were shared. There were relatively no large differences between the two groups in microbial categories. The evolutionary branching diagram of microorganisms showed that most of the genera belonged to Firmicutes, followed by Bacteroidetes. The genus *Akkermansia* belonging to Verrucomicrobia was also found, which is closely related to the health of the gut [39]. Beta diversity analysis of the two groups showed that the samples after fattening were more concentrated and the similarity between the individuals was higher. Large activity area and different feeding conditions of Wenchang chickens before fattening (the free-range breeding) resulted in obvious different composition of microorganisms among individuals. While after fattening in the cages, the feeding condition was relatively concentrated, same diet was maintained, and the similarity in microorganism composition was improved.

Before and after fattening, the predominant bacteria in the cecum were Firmicutes and Bacteroidetes, which was similar to the previous studies [5, 16, 33]. The phylum Fusobacteria was a potential biomarker of Wenchang chickens before fattening, which has been reported to be associated with infections in humans [40]. The relative abundance of Cyanobacteria, Spirochaetes, and Tenericutes were higher in the after fattening group, with such a result caused by genera or species differences. The genera more abundant in the before fattening group were Megamonas and Bacteroides, which could degrade complex plant polysaccharides, and the main end product was propionate [36]. A possible reason was that the free-range breeding before fattening gave Wenchang chickens more opportunities to peck on the plants. However, further work is required to thoroughly understand the role of these cecal bacteria in Wenchang chickens.

Through functional annotations, carbohydrate metabolism was the main metabolic pathway in cecal microbiota of chickens [16], followed by many sequences that were predicted to be related to amino acid and glucose metabolism. “Digestive system” and “Biosynthesis of other secondary metabolites” were two highly enriched functional annotations in Wenchang chickens before fattening by KEGG. Studies on the composition and function of cecal microbiota in Dagu chicken under two feeding modes, free-range (outdoor, OD) and cage (indoor, ID) raising, also found that these two functional pathways were significantly higher in 12 weeks old and 18 weeks old free-range chickens than in the caged chickens [16]. Five functional clusters were annotated by COGs between both the groups. Metabolic functions involved in chickens’ gut microbiota and these functions might vary because of the different compositions of gut microbiota. The chicken meat with free range raising has more protein content and a better water-holding capacity which can improve the meat quality [16, 41]. Addition of probiotics to chicken feed could also improve the meat quality and increase the output of breast and leg muscles [42]. Fattening stages are also likely to be related to the changes in compositions of gut microbiota, but further scientific research is needed to confirm this yet.

## Conclusions

The composition and function of cecal microbiota in Wenchang chicken before and after fattening were found to be different. These findings provide insights into the roles of the gut microbiota in complex traits and contribute to the development of effective ways for regulating fat accumulation during the production of Wenchang chickens. In-depth study on the functions and interactions of intestinal microbiota can help us develop special probiotics to achieve the expected breeding goals.

## Supporting information

S1 Table Body weight. Ten Wenchang chickens in each group; CC1, 120 days age Wenchang chickens before cage raising; CC2 (day 1), at the beginning of cage raising, CC2 (day 60), at the end of fattening. The means difference is significant at the 0.05 level.
(DOCX)
