# Supplementary material for: Characterization of the cecal microbiome composition of Wenchang chickens before and after fattening
Source: PLoS One. 2019 Dec 5;14(12):e0225692. doi: 10.1371/journal.pone.0225692 (PMC6894782; doi:10.1371/journal.pone.0225692)
Supplement: S1 Table — Ten Wenchang chickens in each group; CC1, 120 days age Wenchang chickens before cage raising; CC2 (day 1), at the beginning of cage raising, CC2 (day 60), at the end of fattening. The means difference is significant at the 0.05 level. (DOCX) [file pone.0225692.s001.docx]

**S1 Table Body weight**

| Group | CC1 | CC2 (day 1) | CC2 (day 60) |
| --- | --- | --- | --- |
| Body Weight(kg) | 1.11±0.096^a^ | 1.14±0.14^a^ | 1.40±0.18^b^ |

Ten Wenchang chickens in each group; CC1, 120 days age Wenchang chickens before cage raising; CC2 (day 1), at the beginning of cage raising, CC2 (day 60), at the end of fattening. The means difference is significant at the 0.05 level.
